# Improving Sleep Among Teachers: an Implementation-Intention Intervention

**DOI:** 10.1007/s12529-022-10069-7

**Published:** 2022-03-01

**Authors:** Laura I. Schmidt, Lisa M. Steenbock, Monika Sieverding

**Affiliations:** Institute of Psychology, Department of Gender Studies and Health Psychology, University of Heidelberg, Heidelberg, Germany

**Keywords:** Sleep duration, Sleep quality, Implementation intentions, Self-monitoring, Theory of planned behavior, Teachers

## Abstract

**Background:**

Insufficient sleep is common among teachers and is associated with diverse health risks. This study aimed to predict intention and sleep duration by applying the Theory of Planned Behavior (TPB) and to examine the effectiveness of an implementation-intention intervention to improve sleep duration and quality.

**Method:**

Sixty-nine teachers (*M* = 36.8 years, *SD* = 10.4) were assigned to an active control group (CG) or intervention group (IG). At baseline, TPB variables were assessed and participants of the IG received instructions to develop implementation intentions to reach the goal of sleeping 8 h on average. During a 3-week intervention period, all participants wore an activity tracker (Fitbit Charge HR) to measure sleep duration and kept diaries to assess sleep quality, physical activity, and stress. After 1 month, a 1-week follow-up, including a booster for the IG, was conducted.

**Results:**

Forty-two percent of variance in sleep duration were explained by control variables, past behavior, perceived behavioral control, and intention. Mixed ANOVAS with baseline covariates found a large main effect with longer sleep duration in the IG. A time x group interaction suggested that only the IG slept significantly longer in the follow-up period compared to the intervention period. For sleep quality, a medium-sized main effect for study group was found and a time x group interaction indicated higher sleep quality in the IG for week 3 and the follow-up.

**Conclusion:**

The TPB was effective in predicting sleep intention and duration. Implementation intentions accompanied by daily monitoring and a booster appear to be promising for improving sleep.

**Supplementary Information:**

The online version contains supplementary material available at 10.1007/s12529-022-10069-7.

## Introduction

Poor sleep has been associated with numerous negative health outcomes and impaired professional and non-professional activities [1–3]. Teaching is a highly stressful occupation [4, 5], and compared to the general population, teachers with high job strain have been found to report poorer sleep [6]. Sleep problems were associated with burnout in a Swedish study on teachers [7], and a prospective Swedish study identified “too little sleep” as the main risk factor for developing a clinical burnout [8]. In a larger-scale German study, about every third teacher (32.5%) suffered from burnout and 17.7% reported suffering from severe strain [9]. Insufficient sleep is not only a risk factor for the well-being and health of teachers themselves. Via processes of emotional transmission, stress resulting from too little sleep can be transferred to the students as well: “A well-slept teacher is a better teacher” ( [10], p. 280). However, getting enough sleep is a challenge in this profession, as morning-school hours dictate early wake-up times in most teachers which can lead to sleep deprivation on weekdays [11, 12]. Therefore, the question arises how teachers can get enough sleep during the week, with “enough” being derived from the recommendations of the National Sleep Foundation with a range of nightly sleep duration from 7 to 9 h among adults [13].

Our study aimed to predict sleep duration among teachers using the Theory of Planned Behavior (TPB; [14] ) and investigated whether an intervention based on implementation intentions can increase sleep hours and sleep quality in a controlled approach. Although the TBP has already been applied successfully in the context of sleep [15], all studies were conducted among students, so that results should not be generalized to our target group of teachers without further examination.

The TPB states that the most proximal determinant of an individual’s behavior is behavioral intention, which is understood as a function of three motivational variables: perceived behavioral control (PBC), subjective norm, and attitude towards the behavior. It is further assumed that PBC can determine behavior directly as well. The TPB has been successfully applied to a wide range of health-related behaviors like physical activity, nutrition behaviors, and alcohol consumption [16–18].

However, the application of the TPB in the study of sleep behavior is surprisingly rare and focusses on student samples. A recent review [15] identified six studies using the TPB measuring actual behavioral outcomes beyond intention in the areas of sleep hygiene, sleep duration, or sleep patterns, and an additional study was published afterwards [19]. Knowlden et al. [20] reported that attitude, subjective norm, and PBC explained 36% of variance in behavioral intention to get enough sleep, and PBC and intention explained 34% of variance in self-reported last night’s sleep duration among US American students. In a second cross-sectional study with Chinese students, TPB predictors explained 43% of variance in the intention to sleep healthily and PBC and intention explained 19% regarding the respective behavior [21]. Among Australian university students, Kor and Mullan [22] found that intention and PBC were predictors of sleep hygiene behavior, although response inhibition (Go/NoGo task) accounted for more variance. Two studies assessed self-reported and actigraphy-recorded sleep duration among US students over the course of 1 week [23, 24]. The first indicated that intentions predicted variability in self-reported (18%) and actigraph sleep duration (14%), with PBC predicting 9% of additional variability for both measures beyond intention [23]. Similarly, the second [24] reported that intention significantly explained diary-based (15%) and actigraphy-recorded sleep duration (11%). The study with the largest follow-up period of 6 months examined sleep hygiene and quality of Iranian high school students [25]. TPB predictors explained about 50% of variance in behavioral intentions regarding sleep hygiene. Intention, PBC, coping and action planning together accounted for 60% of variance in sleep hygiene behavior, which, in turn, explained about 8% of variance in sleep quality. The most recent prospective study [19] on sleep hygiene in Australian and Hong Kong university students pointed out that TPB models including action planning explained 52% (Australia) and 48% (Hong Kong) of variance in sleep hygiene behavior assessed 4 weeks after a baseline questionnaire.

### Implementation Intentions

The key variable in the TPB is the intention to behave in a certain way, in this case, the intention to get enough sleep. However, as in other areas of health-related behavior, there often is a gap between intention and behavior [26]. As Loft and Cameron stated, many working adults have the intention to get sufficient sleep “but they fail to execute the necessary actions to do so” (27, p. 260). An effective method to overcome the intention-behavior gap is the use of implementation intentions: if–then plans that link anticipated situations to specific goal-directed responses. Thereby, behaviors become more automatic, as the need for conscious decision-making and distraction is reduced by the mental stimulus–response linkage between cue and behavior [28]. A meta-analysis including 94 studies calculated that implementation intentions had a positive effect (*d* = .65) on goal attainment [29]. Up to now, implementation intentions were only very rarely applied in intervention studies aiming to improve sleep. Two studies have yielded promising results in improving sleep patterns by building easy but effective “if–then” structured intentions [27, 30]. A third recent study found positive effects of implementation intentions combined with mental contrasting on bedtime procrastination [31] in terms of a reduced discrepancy between intended and actual bedtime. However, self-reported sleep duration was not affected.

All three studies used sleep diary methods that are more reliable than aggregated retrospective ratings. However, as certain parameters such as time of falling asleep or frequency of nighttime awakenings may not be sufficiently accurate because of low levels of consciousness, the assessments of objective sleep parameters could serve as a valuable complement to daily diaries.

### Study Aims and Hypotheses

The present study assessed subjective and objective sleep outcomes in German teachers using an activity tracking device, the Fitbit Charge HR, and daily sleep diaries. Our first aim was to examine the predictive value of the TPB with regard to sleep intention and duration, as previous studies have concentrated on student samples. Our second aim was to investigate whether an intervention based on implementation intentions is effective in improving sleep outcomes. The core of our intervention was providing face-to-face guidance to develop individualized if–then plans. As recent health psychological research identified the importance of booster sessions or interventions for the efficacy and maintenance of effects in health contexts (i.e., [32, 33] ), a booster after a break of 1 month was included in a follow-up week.

Regarding our first aim, we hypothesized that the TPB variables attitude, subjective norm, and PBC predict the intention to sleep approximately 8 h per night at baseline (H1) and that intention and PBC predict sleep duration during the intervention period (H2). Regarding our second aim, we hypothesized that during the intervention period and during the follow-up, the intervention group (IG) receiving an implementation-intention intervention would exhibit longer sleep duration (H3) and higher subjective sleep quality (H4) compared to an active control group (CG).

## Method

Ethical approval was obtained by the ethics commission of the Faculty of Behavioural and Cultural Studies at Heidelberg University (protocol number: AZ Schm 2018/1–1).

### Recruitment and Sample

Teachers were recruited at a private educational institute which runs several general schools and institutes at different locations in two larger German cities. The study was presented during staff meetings and via mailing lists. The following exclusion criteria were specified: [1] suffering from insomnia (DSM-5 diagnostic criteria), (2) taking prescribed insomnia medication, (3) working less than 20 h as a teacher. To avoid communication between participants of the CG and the IG, we chose not to randomly assign single participants to each study group, but followed a quasi-experimental design with the advantage that participants were allowed to talk about the study but did not know about the other condition. Thereby, participants were allocated to the two study groups alternatingly in teams by workplaces (= 6 schools). In detail, the first teachers from schools 1, 3, and 5 who registered were allocated to the IG, as were all her/his colleagues from the same sites that registered later during recruitment. The respective first counterparts and colleagues from schools 2, 4, and 6 were allocated to the CG.

**Fig. 1:**
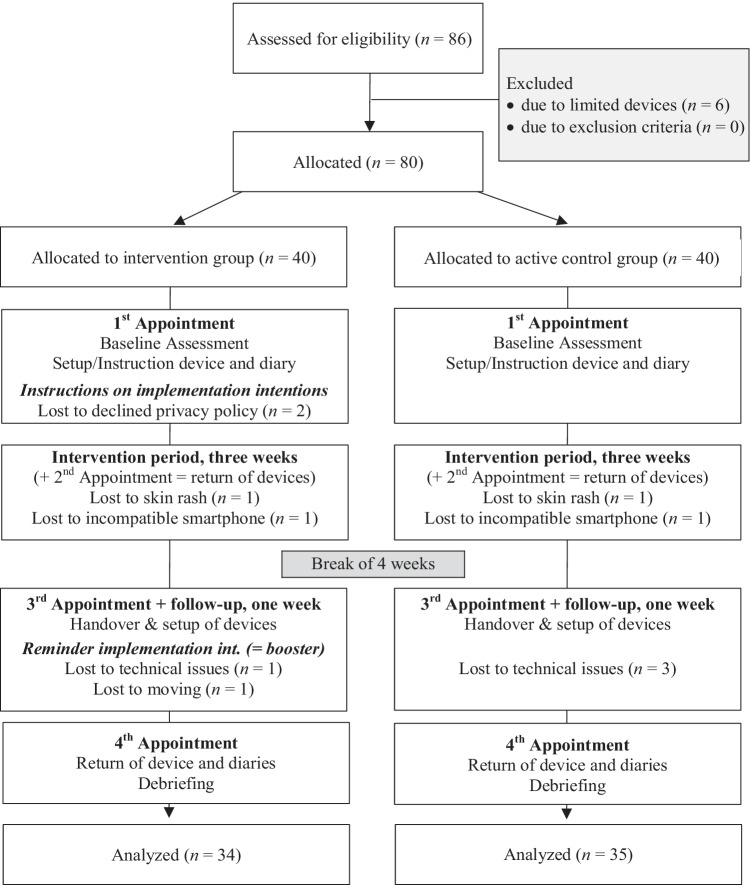
Participant flow diagram

Initially, we included 80 teachers in the study, of whom two persons decided not to further participate because of the privacy policy of Fitbit, Inc. after the first appointment. We lost data of six participants due to technical problems (i.e., incompatible smartphone, data transmission faults). Due to skin rash, two participants had to stop wearing their device and one participant moved during the survey period and did not participate in the follow-up (see Fig. 1). The final sample consisted of *N* = 69 teachers aged 24 to 70 years (*M* = 36.8 years, *SD* = 10.4). Fifty were female, 54 lived in a relationship or marriage, and 14 had at least one child. On average, teachers reported working 41.0 h per week (*SD* = 8.4).

### Procedure and Content of the Intervention

The study consisted of four appointments with the research manager (L.M.S.) which were held in different facilities of the educational institute (separated for study groups, see Fig. 1). Participants were told that the overall objective was either monitoring of several health parameters like physical activity, stress, and sleep (CG) or the improvement of sleep (IG). Before they provided informed consent, participants were given an opportunity to ask questions. At the first appointment, all participants received information on the procedure of the study and the Fitbit, and completed the questionnaires assessing demographics and TPB variables (Fig. 1). Each participant was guided through the setup of the Fitbit and connection with the smartphone. The intervention group additionally received the implementation-intention intervention in the form of a worksheet that contained (1) information on the NSF sleep recommendation (7–9 h), (2) anticipation of typical individual barriers and guidance on how to plan sleep and bedtime routine with a timetable, and (3) introduction of the concept of implementation intentions and concrete examples (whenever/if–then structure). Subsequently, participants were instructed: “Please formulate at least three implementation intentions in order to reach the goal to sleep eight hours on average. Think about your individual barriers or consider preventive measures that could help you get enough sleep.” Examples specified by the participants were as follows: “Whenever I have finished eating dinner, I prepare my lunch bag and my working bag for tomorrow,” “Whenever I correct tests and assignments in the evening, I set my alarm to stop at nine p.m.,” or “Whenever the time is 30 min before my intended bed time, I switch off my phone.” For the following 3-week intervention period, both groups were asked to record subjective stress, active minutes, steps (Fitbit), sleep quality, and sleep duration (Fitbit) in their daily diaries. Participants of the IG additionally were reminded to re-formulate their if–then plans if the intended sleep duration was not achieved. At a second appointment following the 3-week intervention period, participants returned the diaries and the devices. After a break of 4 weeks, the follow-up started. For this, all participants again received a Fitbit at a third appointment and answered analogous diaries for seven consecutive days including identical reminders to re-formulate the if–then plans (if necessary) only in the intervention group (= booster). At the fourth appointment that ended the follow-up, a final questionnaire on usability and perceived influence regarding the monitoring was filled out. All participants were debriefed about the aims of the study and were offered to receive a summary of results via e-mail.

### Measures

#### Pilot Questionnaire

Following Fishbein and Ajzen’s [34] guidelines of TPB questionnaire construction, we administered a pilot questionnaire to 12 individuals from different professions that did not take part in the main study (66.6% female, age: *M* = 35.8, *SD* = 4.5, range 26 to 55 years) for the purpose of item generation. To elicit behavioral beliefs, the participants were asked to name advantages and disadvantages of sleeping 8 h on average. To elicit normative referents, they were asked to list all individuals or groups agreeing that they should sleep 8 h on average. To elicit relevant influencing variables, they were asked to list all factors enhancing and hampering sleeping 8 h on average. Answers were used for the construction of the final questionnaire, which included both direct and indirect measures using the salient beliefs of the pilot questionnaire. Based on Fishbein and Ajzen’s [34] guidelines, the target behavior, namely sufficient sleep duration, was defined as an average sleep duration of 8 h for the next 3 weeks in both the pilot and the final questionnaires. All items are available on request. As direct measures outperformed indirect measures in terms of variance explanation, we only report the items and scales belonging to the direct measures that were used in our analyses for this present work. For details regarding perceived usability of the Fitbit device, see Electronic Supplementary Material (ESM) 1.

#### Assessment of the Theory of Planned Behavior (TPB)

For all items, if not specifically mentioned, 7-point Likert scales ranging from 1 (“strongly disagree”) to 7 (“strongly agree) were used. As past behavior has been recognized as an important control variable in the application of TPB models [22, 35], *past sleep duration* was measured using one item, namely “Please estimate how many hours you slept per night on average for the past four weeks.” *Attitude* was measured using ten 7-point semantic differential scale items with the prompt: “To sleep on average 8 h during the next three weeks would be…” (i.e., unhealthy–healthy, unpleasant–pleasant, unimportant–important; Cronbach’s *α* = .87). *Subjective norm* was assessed with two items (“People who are important to me think / my doctor thinks…I should sleep for eight hours on average”; Cronbach’s *α* = .88). *Perceived behavioral control (PBC)* was assessed using two items, namely “I am sure that I will be able to sleep 8 h on average for the next three weeks” and “It is up to me if I will be able to sleep 8 h on average for the next three weeks” (Cronbach’s *α* = .68). *Behavioral intention* was measured using two items adapted from Sieverding et al. [36], namely, “I intend to sleep 8 h on average for the next three weeks” and the subjective probability for realizing this goal (“How likely is it in percent from 0 to 100% that you will sleep 8 h on average for the next three weeks?” Cronbach’s *α* = .76). To calculate the mean score with possible scores from 0 to 6, intention items were aggregated using the formula ((item 1 − 1) + (item 2*6/100))/2 following previous studies [36, 37].

#### Diary Variables: Sleep, Physical Activity, and Stress

*Sleep duration*, as the primary outcome measure, was assessed via Fitbit and transferred by participants to their daily diary. To capture the secondary outcome *subjective sleep quality*, two items of the Pittsburgh Sleep Diary (Monk et al. 1994) were used in a slightly modified version. Participants were asked to rate how well they slept subjectively and how rested they felt when waking up on a scale ranging from 0 (“very bad”) to 100 (“very good”; *α* = .86). If participants slept less than intended, they were asked to name reasons regardless of the group, while only the intervention group was subsequently asked to form a new or altered implementation intention addressing the respective reason. Further insights regarding categories and frequencies of those reasons recorded in the daily diaries are given in Electronic Supplementary Material (ESM) 2. Daily physical activity measures included *number of steps*, copied to the diary from the Fitbit application, and the self-reported duration of *moderate to intense activity (“active minutes”)*. To achieve a comparable effort, the CG was asked to name reasons, why, if the case, they did not engage in physical activity. Daily *stress* was assessed using one item from the HEI-STRESS [38] ranging from 0 (“not at all”) to 100 (“very stressed”).

### Statistical Analyses

As three previous studies applying implementation intentions to improve sleep (hygiene) outcomes yielded promising results and about medium effect sizes [27, 30, 39], we used this as an estimation for our power analysis (G*Power; [40] ). Assuming a one-sided test with a 5% *α* level and power of 80%, the required sample size was 60. With an anticipated 10–15% dropout rate, for example, due to technical problems, the aim was to recruit 70 participants. This sample size would also be enough to detect medium-sized effects in linear multiple regressions (G*Power: fixed model, *R*^2^ increase, H1: 3 tested predictors, 5 total, *f*^2^ = .17; H2: 2 tested predictors, 6 total, *f*^2^ = .15; H3: 1 tested predictor, 7 total, *f*^2^ = 0.12). Statistical analyses were performed using SPSS version 24.0. The Kolmogorov–Smirnov tests revealed normal distribution of aggregated sleep parameters (duration and quality), whereas intention and PBC showed violations. Examinations of skewness and kurtosis then revealed low coefficients (PBC: skewness =  −0.49, kurtosis =  −0.92; intention: skewness =  −1.00, kurtosis = 0.14) that have been reported to produce robust results in Monte Carlo simulations [41]. In preliminary analyses, we used *t*-tests to examine group differences in baseline variables in order to control for possible differences in subsequent analyses. Key assumptions regarding regression analyses were met (linear relationship, multivariate normality, no multicollinearity or auto-correlation, homoscedasticity). With respect to the first hypotheses, two regression analyses on behavioral intention (H1) and sleep duration (H2) were conducted followed by relative weight analyses (RWA) which allow partitioning the explained variance among multiple predictors [42], as opposed to the indices commonly produced by multiple regression which fail to appropriately partition variance to correlated predictors. As robust evidence indicates reduced sleep duration among women and older (but still working) individuals [43], as well as negative effects of daily stress [44], we a priori included age, gender, and daily stress as covariates in the regression analyses predicting sleep duration. With respect to Hypotheses 3 and 4, mixed 2 (group) × 4 (time) ANOVAS including covariates were used to analyze repeated measures of sleep duration and quality. Normality was checked using the Shapiro–Wilk test, which indicated that for nearly every measurement period, except for the intervention group in week 3, sleep durations follow normal distribution. Regarding sleep quality, the normality assumption was slightly violated for the intervention group in the follow-up period only. Consequences of similar failures to meet normality assumptions have been investigated in a meta-analysis reporting the robustness of the *F*-test in ANOVAS [45]. Sphericity assumptions were met (Mauchly tests, *p*’s > .05) and homogeneity of error variances and covariances was given, as assessed by Levene’s tests and Box’s tests (*p*’s > .05). With 69 participants completing the study and 28 nights measured, the missing rate for the total of 1932 nights was only 7% regarding sleep duration and quality. Missing information was not related to sociodemographic data or TPB variables. As missing rates were relatively low, values were not imputed [46].

## Results

### Explaining the Intention to Sleep 8 h with TPB Variables at Baseline (H1)

Table 1 depicts descriptive statistics and correlations between study variables. Participants with more positive attitudes, higher subjective norms, and higher PBC reported a higher intention to sleep 8 h on average. In a multiple linear regression analysis (*adjR*^2^_cum_ = .51) followed by a relative weight analysis, age (*β* = .05, *p* > .05, *RW* = 0.7), gender (*β* = .15, *p* > .05, *RW* = 1.9), and subjective norm (*β* = .09, *p* > .05, *RW* = 5.6) did not significantly contribute to variance explanation, while attitudes (*β* = .49, *p* < .001, *RW* = 50.4) and PBC (*β* = .39, *p* < .001, *RW* = 41.3) were significant predictors of intention. Hence, Hypothesis 1 could be confirmed, with the TPB variables explaining 51% of variance in behavioral intention, corresponding to a large effect (*f*^2^ = 1.04).

**Table 1:** Descriptive statistics and intercorrelations of the study variables for the total sample

Variables	*M*	*SD*	2	3	4	5	6	7	8	9	10	11	12	13
1 Gender^a^	–	–	.09	.15	.20	−.17	.10	−.02	.09	.16	.03	−.01	.13	.01
2 Age	36.77	10.39		.30*	−.21	−.15	−.05	−.10	−.15	−.01	.25*	.04	−.05	−.01
3 Workload^b^	41.01	8.42			.01	−.04	−.09	−.11	−.13	.01	.08	.06	−.19	−.17
4 Attitude^c^	5.07	.92				.25*	.39*	.63**	.48**	.49**	−.04	−.06	.08	−.08
5 Subjective norm^c^	4.33	1.61					.06	.25*	.13	.04	−.23	.27*	.00	.15
6 Perceived behavioral control^c^	4.59	1.55						.57**	.44**	.39*	.29*	−.40*	.04	.23
7 Intention^d^	3.96	1.51							.54**	.56**	.04	−.20	.11	.14
8 Past sleep duration^e,f^	7.01	.65								.59**	.22	−.23	.16	.19
9 Fitbit-measured sleep duration^f^	7.36	.58									.25*	−.28*	.05	.05
10 Self-reported sleep quality^g^	64.22	11.71										−.45**	.19	.27*
11 Self-reported stress^h^	40.80	13.86											−.03	−.06
12 Steps per day (Fitbit)	10,186.99	2177.40												.47**
13 Active minutes per day^i^	36.02	23.55												–

*N* = 69. Variables 1 to 8 were assessed at baseline; variables 9 to 13 were aggregated over the intervention period (3 weeks)

Correlation patterns were very similar if broken down per study group; a separated correlation table is available on request

^a^50 women (72%), 0 = male, 1 = female

^b^Number of working hours per week, range 20 to 50 h

^c^From 1 to 7 higher scores indicating stronger agreement

^d^From 0 to 6, higher scores indicating a higher intention to sleep 8 h per night

^e^Self-report (last 4 weeks)

^f^In decimal hours

^g^From 0 to 100 (“very good”)

^h^From 0 to 100 (“very stressed”)

^i^Self-reported moderate to vigorous physical activity

^*^p < .05; **p < .001

### Prediction of Sleep Duration with TPB Variables (H2)

Sleep duration during the intervention period was positively associated with PBC, intention, and daily stress, but did not correlate with active minutes and steps per day (see Table 1). A hierarchical regression model explaining (fitbit-measured) sleep duration during the intervention period is presented in the left part of Table 2, with relative weights (*RW*) indicating the respective amount of explained variance per predictor. Past behavior (self-reported sleep duration over the last 4 weeks) was entered in the first step. Age, gender, and daily stress were entered in the second, and PBC and intention to sleep 8 h in the third step. The overall model explained 42% of variance in sleep duration (*adjR*^2^_cum_), corresponding to a large effect (*f*^2^ = .72). The addition of intention and PBC at step 3 increased the variance accounted for by 8%, confirming H2. According to RWA, intention was 3.9 times (35.8/9.2) more important than PBC as a predictor of sleep duration.

**Table 2:** Hierarchical regression analyses predicting fitbit-measured sleep duration in the intervention period by TPB variables

*Aggregated sleep duration (fitbit-measured)*^*a*^												
Step	Predictor	*β*_step 1_	*β*_step 2_	*β*_step 3_	***RW%***^**b**^	Step	Predictor	*β*_step 1_	*β*_step 2_	*β*_step 3_	*β*_step 4_	***RW%*** ^**b**^
1	Past sleep duration^c^	.59**	.56**	.38*	41.7	1	Past sleep duration^c^	.59**	.56**	.38*	.40**	35.6
2	Age		.08	.08	0.7	2	Age		.08	.08	.05	0.4
	Gender^d^		.11	.13	4.5		Gender^d^		.11	.13	.11	3.2
	Stress^e^		−.16	−.14	8.1		Stress^e^		−.16	−.14	−.11	5.7
3	Perceived behavioral control^f^			−.06	9.2	3	Perceived behavioral control^f^			−.06	−.05	6.9
	Intention^f^			.37^*^	35.8		Intention^f^			.37*	.24	23.3
						4	Study group^g^				.30*	24.8
Δ*R*^2^		.35**	.04	.08*				.35**	.04	.08*	.07*	
*AdjR*^2^_cum_		.34	.35	.42				.34	.35	.42	.49	

*N* = 69; Method = Enter

^a^Intervention period: aggregated sleep duration over 3 weeks

^b^*RW%*, relative weights; relative contribution (in percentage) of each predictor to the total explained variance (*R*^*2*^)

^c^Retrospective self-reported sleep duration at baseline (last 4 weeks)

^d^0 = male, 1 = female

^e^Self-reported stress (daily diary); possible values from 0 to 100 (“very stressed”)

^f^Variables of the Theory of Planned Behavior (TPB), higher scores indicating higher agreement

^g^0 = active control group, 1 = intervention group

Interactions were tested but did not explain further variance; *p < .05; **p < .001

### Effects of the Implementation-Intention Intervention on Sleep Duration (H3)

To address our second aim regarding the effectiveness of the intervention, we first repeated the regression analysis on overall sleep duration with the variable study group in a fourth step. We thereby aimed to explore the share of variance that can be attributed to (baseline) TPB variables vs. the effect of our intervention that should be illustrated in the assignment to our two study groups. This second model explained 49% of variance in sleep duration (see right part of Table 2; *f*^2^ = .96). Adding study group in the 4^th^ step significantly increased explained variance in sleep duration over and above past behavior, control variables, and TPB variables (accounting for 7% incremental variance). According to RWA, intention and PBC together accounted for 30.2% and study group for 24.8% of explained variance.

In further preliminary analyses before testing the effects of our intervention in mixed ANOVAS, we compared baseline scores of IG and CG. As *t*-tests revealed higher scores in PBC (*t*(67) =  −2.12, *p* < .05) and intention (*t*(67) =  −3.53, *p* < .01) among teachers assigned to the IG, our further analyses took into account those baseline differences by controlling for those variables. Study groups did not differ in any other baseline variables, i.e., (demographics, past sleep duration, further TPB constructs, stress, and physical activity).

**Fig. 2:**
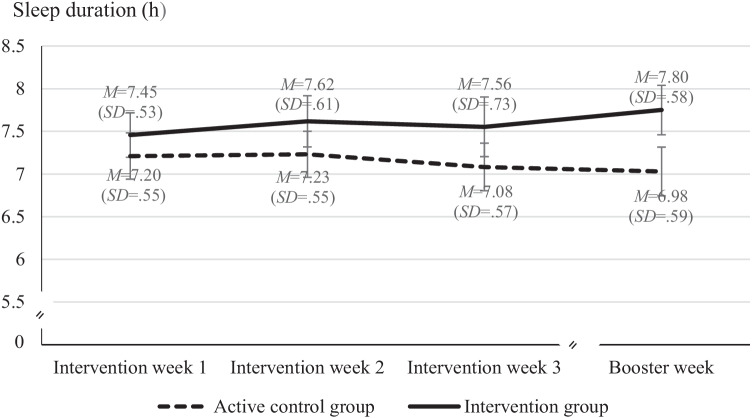
Fitbit-measured sleep duration as a function of study group and time after intervention. Mixed ANOVA with baseline scores for perceived behavioral control and intention as covariates; intervention period, weeks 1–3: aggregated fitbit-measured sleep duration from 7 consecutive days; follow-up period (booster week) after a break of 4 weeks: aggregated fitbit-measured sleep duration from 7 consecutive days. Error bars represent one standard deviation. Significant group differences in sleep duration for intervention weeks and follow-up (Welch tests; *p* < .001); Cohen’s *d*_(week 1)_ = .84, 95% CI [.35; 1.33]; Cohen’s *d*_(week 2)_ = .93 [.44; 1.43]; Cohen’s *d*_(week 3)_ = 1.06 [.56; 1.56]; Cohen’s *d*_(follow-up)_ = 1.41 [.89; 1.94]. Mean group differences and CI (in minutes): *M*_Diff(week 1)_ = 27.1, CI [11.5; 42.6]; *M*_Diff(week 2)_ = 32.6, CI [15.8; 49.4]; *M*_Diff(week 3)_ = 41.8 CI [22.9; 60.7]; *M*_Diff(week 4)_ = 49.6, CI [32.6; 66.7]

As assumed in Hypothesis 3, large effect sizes in fitbit-measured sleep duration were observed favoring the IG in the intervention period (IG: *M* = 7.64 h, *SD* = .55; CG: *M* = 7.08 h, *SD* = .48; *t*(67) =  −4.57, *p* < .001; Cohen’s *d* = 1.10) and the follow-up (IG: *M* = 7.80 h, *SD* = .58; CG: *M* = 6.98 h, *SD* = .59; *t*(66) =  −5.82, *p* < .001; *d* = 1.41). In order to investigate how the intervention affected sleep duration during the course of the study, a mixed ANOVA with fitbit-measured sleep duration as dependent variable (aggregated scores for intervention week 1, intervention week 2, intervention week 3, and the follow-up week) was conducted. Baseline scores in PBC and intention were entered as covariates. Results revealed a large main effect for study group with longer sleep duration in the IG (*F*(1, 64) = 14.03, *p* < .001, partial *η*^2^ = .18) and a medium-sized main effect for baseline scores in intention (*F*(1, 64) = 6.95, *p* < .05, partial *η*^2^ = .10). Moreover, there was a significant interaction between time and group (*F*(3, 192) = 4.41, *p* < .01, partial *η*^2^ = .06) (see Fig. 2), and a significant effect of time on sleep duration in the IG (*F*(3, 93) = 4.89, *p* < .01, partial *η*^2^ = 014), but not in the CG (*F*(3, 93) = 0.51, *p* > .05, partial *η*^2^ = .02). Participants in the IG slept significantly longer than their counterparts in the CG during the three intervention weeks and during the follow-up (Welch tests; *p* < .001; Cohen’s *d*s, mean differences, and confidence intervals, see Fig. 2), as assumed in Hypothesis 3. Pairwise comparisons indicated a significant difference between week 1 and follow-up only in the IG (*p* < .05), with longer sleep duration at follow-up.

### Effects of the Implementation-Intention Intervention on Self-reported Sleep Quality (H4)

**Fig. 3:**
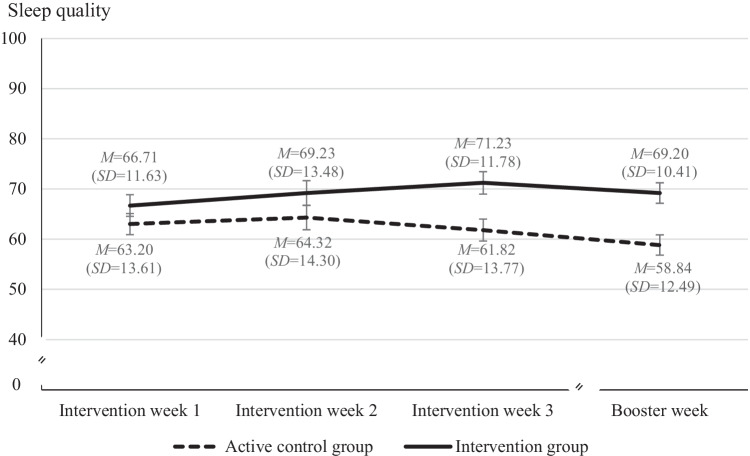
Self-reported sleep quality as a function of study group and time after intervention. Mixed ANOVA with perceived behavioral control as covariate; sleep quality: daily self-report (2 items); from 0 (“very bad”) to 100 (“very good”), *α* = .86; intervention period, weeks 1–3: aggregated sleep quality from 7 consecutive days; follow-up (booster week) after a break of 4 weeks: aggregated sleep quality from 7 consecutive days. Error bars represent one standard deviation. Significant group differences in sleep quality for intervention week 3 and follow-up week (Welch tests; *p* < .01); Cohen’s *d*_(week 1)_ = .29, 95% CI [−.18; .77]; Cohen’s *d*_(week 2)_ = .27 [−.21; .74]; Cohen’s *d*_(week 3)_ = .71 [.22; 1.19]; Cohen’s *d*_(follow-up)_ = .75 [.26; 1.23]. Mean group differences and CI: *M*_Diff(week 1)_ = 3.7, CI [−2.4; 9.8]; *M*_Diff(week 2)_ = 3.7, CI [−2.9; 10.4]; *M*_Diff(week 3)_ = 9.1, CI [2.9; 15.2]; *M*_Diff(week 4)_ = 8.6, CI [3.0; 14.1]

For sleep quality, a second mixed ANOVA with analogously aggregated scores for each intervention week and follow-up week and PBC as covariate was conducted. Results indicated a significant interaction between time and group (*F*(3, 198) = 3.02, *p* < .05, partial *η*^2^ = .04) with Welch tests demonstrating higher sleep quality in the IG for week 3 and the follow-up (*p* < .01; for Cohen’s *d*s, *M*, *SD*, mean differences, and confidence intervals, see Fig. 3). A second interaction between time and PBC indicated that participants with higher scores in PBC reported higher sleep quality during the intervention period but not during the follow-up (*F*(3, 198) = 4.27, *p* < .01, partial *η*^2^ = .06). There was a small- to medium-sized main effect for study group with higher sleep quality in IG (*F*(1, 66) = 4.2, *p* < .05, partial *η*^2^ = .06) confirming Hypothesis 4.

## Discussion

The first aim of our study was to evaluate the usefulness of the TPB to predict behavioral intention (H1) and fitbit-measured sleep duration (H2) among teachers. The intention to sleep 8 h on average could be explained by attitudes and PBC, whereas subjective norm did not contribute to variance explanation. With respect to fitbit-measured sleep duration, the TPB variables intention and PBC together accounted for 45% of explained variance (RW) over and above the effect of self-reported past sleep duration.

The second aim was to explore the effectiveness of an intervention based on implementation intentions on sleep duration (H3) and quality (H4). For fitbit-measured sleep duration, mixed ANOVAS revealed a main intervention effect (*η*^2^ = .18); participants of the intervention group slept significantly longer than participants of the active control group; large effect sizes were found for the three intervention weeks and the follow-up. Moreover, we found a main effect of baseline intention scores, and an interaction of time and group indicated longer sleep duration at follow-up, which included the booster, compared to intervention week 1 only in the intervention group. For self-reported sleep quality, an interaction of time and group indicated significantly higher sleep quality in the intervention group for week 3 and the follow-up; the main effect for group was small to medium (*η*^2^ = .06).

Effect sizes were larger for sleep duration, which was the main target of our intervention. Participants were instructed to formulate implementation intentions that were useful to increase this main outcome parameter and did so (i.e., stop working at a certain time and prepare for bed), but some of their implementation intentions also addressed sleep hygiene behaviors that might also help to increase sleep quality (i.e., not using the mobile phone in bed/before sleeping). As indicated in Table 1, sleep duration and quality were significantly but only modestly correlated (*r* = .25; *p* < .05). As several studies show that the effects of sleep duration and sleep quality on health outcomes are not simply additive, it is crucial to consider both facets where possible [47].

Our findings are partly in line with those of Mairs and Mullan [30] who reported isolated positive effects of implementation intentions for improving self-reported sleep outcomes, measured with the Pittsburgh sleep quality index (PSQI) and the insomnia severity index (ISI) in university students. Unlike Mairs and Mullan [30], we did not find improvements through self-monitoring only. Alongside differences in study populations, this might also be due to the fact that the monitoring task in our active control group was not specifically tailored to sleep parameters, but rather had a broader health focus (physical activity, stress, and sleep), which may have shifted the focus of attention. However, our results are in line with the randomized controlled trial of Melton et al. [48] on the usage of an activity tracking device for monitoring purposes that found no changes in sleep duration and sleep efficiency. In contrast to our study, Valshtein et al. [31] did not find effects of implementation intentions combined with mental contrasting on sleep duration among students, which might be due to a higher baseline sleep duration reported in that study.

Variance explanation by TPB variables regarding the intention to sleep 8 h on average (*R*^2^ = .51) was comparable or even higher than in studies on student samples (*R*^2^ = .36, *R*^2^ = .43, and *R*^2^ = .50) [20, 21, 25], although it has to be taken into account that the framing of goal intentions varied (i.e., “enough sleep,” “sleep healthily,” “practice sleep hygiene behavior”). In contrast, subjective norm did not contribute to variance explanation in intention in our sample of teachers. This may be due to a higher importance of normative influences (i.e., by peers) in student populations compared to teachers. Moreover, we found that PBC was a strong predictor for intention. The finding that PBC did not emerge as a significant predictor in the regression analyses on sleep duration was partly due to the inclusion of past sleep duration (correlation with PBC: *r* = .44), which is in line with findings of Kor and Mullan [22] that support evidence on the predictive power of past behavior. However, PBC revealed significant bivariate associations with fitbit-measured sleep duration (*r* = .39) and sleep quality (*r* = .29). Compared to other occupations, teachers report a higher effort-reward imbalance and overcommitment [5, 49], which relates to our finding that the most frequent reason for insufficient sleep was “working too long” (see ESM 1). Future interventions should target those aspects alongside self-efficacy and implementation intentions.

### Strength and Limitations

To the best of our knowledge, the present study is the first in the context of sleep behavior that combines the established framework of the TPB and an implementation-intention intervention with ambulatory assessment in a longitudinal design. Additional strengths include a non-student sample, a strong control condition, the inclusion of a booster for the intervention group during follow-up, a standardized intervention protocol allowing replication of the results, and the combined collection of self-report and fitbit-based data over a longer time period. According to the recommendation of the German Association for sleep research and sleep medicine [50], sleep diaries should be completed for at least 14 days to receive valid data. In our study, sleep and activity diaries were filled out for 3 weeks (intervention period) plus one follow-up week alongside the fitbit-measured variables.

However, several limitations should be considered. First, randomization of single persons was not possible as participants were allocated into the two study groups in teams by workplaces. Thereby, we avoided communication between participants of the CG and the IG, but effects of external characteristics may still remain, although baseline differences were taken into account and groups did not differ in terms of past sleep behavior, demographics, stress level, and physical activity. However, threats to internal validity due to the quasi-experimental design cannot be ruled out [51]. Second, the findings are based on a relatively small sample in a rather homogeneous population of teachers who have to get up early, which limits sleep duration if not in bed on time. Studies with larger numbers of (heterogeneous) non-student participants are needed, as findings may only be valid for populations with comparable job-related characteristics. Third, a condition with implementation intentions but without the self-monitoring component would have been useful to isolate the effects of planning. Fourth, as social-cognitive predictors such as self-efficacy were only measured at baseline, we were not able to depict possible changes and derive underlying mechanisms of action. Fifth, past sleep duration was only measured via retrospective self-report that might be affected by recall bias and we did not assess past sleep quality. Sixth, according to a recent meta-analysis [52], newer generations of sleep-staging Fitbit models (i.e., Fitbit Alta 2) outperform early-generation models in terms of sensitivity and specificity. In reference to polysomnography, nonsleep-staging models such as the Charge HR have the tendency to overestimate total sleep time, whereas for sleep-staging models, no significant difference was found. More general, the Fitbit devices cannot be recommended for accurate point estimates, but have demonstrated the ability to correctly identify the effects of interventions in validation studies [53]. Respective interventions using rather affordable commercial devices (vs. cost-intensive research sensors) may facilitate transfer into practice.

### Conclusion and Outlook

In sum, our results support our proposition that the Theory of Planned Behavior is a useful framework for the prediction of sleep intention and duration in a non-student sample. Hence, TPB constructs should be included in further research and can be promoted through a training intervention, e.g., via self-efficacy with effects on PBC and intention. Further, our findings complement research on sleep interventions by providing ambulatory-assessed data, with an implementation-intention intervention yielding promising effects. The inclusion of a booster follow-up starting 7 weeks after the first appointment had beneficial effects within the intervention group, as the effects on both sleep duration and quality intensified during this period. However, larger sample sizes are needed to additionally test the effectiveness of a booster vs. no booster condition in a between-subjects design with respective subgroups in the intervention group. Longer follow-up periods are needed to determine whether such effects can be maintained over time, and if additional support (e.g., volitional help sheets [54]) is needed. For efficient design of behavior change interventions, more studies are needed to disentangle the effects of the “ingredients” and address the question if there are additive or multiplicative effects of distinct components. According to the taxonomy of Michie et al. [55] that identified a 16-cluster solution, our intervention components can be classified into the clusters “goals and planning” and “feedback and monitoring.” Over and above the effects of behavior change techniques, further studies should consider moderators, mediators, and secondary outcomes that have been recently linked to self-regulation and sleep outcomes and might be important for teachers as well, i.e., sleep hygiene behavior, chronotype, the tendency to engage in bedtime procrastination, detachment from work, perseverative cognitions, and burnout [56–59]. Moreover, it might be even more effective to tailor the intervention (and assessment of intentions) towards individual sleep need instead of using a generalized goal of sleeping 8 h. Such an approach would take existing interindividual differences in sleep need into account [60]. To conclude, our study contributes to behavior change literature through the development and testing of a theory-guided intervention targeting sleep behavior among teachers. Further research should address efficacy of similar interventions to improve sleep in a greater variety of populations.

## Supplementary Information

Below is the link to the electronic supplementary material.

Supplementary file1 (DOCX 30 KB)

Supplementary file2 (DOCX 31 KB)
