# Supplementary material for: Improving Sleep Among Teachers: an Implementation-Intention Intervention
Source: Int J Behav Med. 2022 Mar 1;30(1):49–61. doi: 10.1007/s12529-022-10069-7 (PMC9879815; doi:10.1007/s12529-022-10069-7)
Supplement: Supplementary file 1 — Supplementary file1 (DOCX 30 KB) [file 12529_2022_10069_MOESM1_ESM.docx]

Electronic Supplementary Material 1:

***Usability and Satisfaction***

We assessed usability regarding the Fitbit device with four items (e.g., “wearing the Fitbit is comfortable”, “the Fitbit is easy to use”; Cronbach`s α = .60) adapted from the Telehealthcare Satisfaction Questionnaire - Wearable Technology ([TSQ-WT; 1](#_ENREF_1)). All items were rated on a 5-point scale ranging from 0 (“not at all”) to 4 (“completely right”). Mean satisfaction with the Fitbit device assessed with TSQ-WT items was high (*N* = 69; *M* = 3.3, *SD* = .54).

Reference:

1. Zijlstra W, Clemens B, Klaus P. Wearable systems for monitoring mobility related activities: From technology to application for healthcare services. In: Carsten R, Martina Z, editors. E-Health, Assistive Technologies and Applications for Assisted Living: Challenges and Solutions. Hershey, PA, USA: IGI Global; 2011. p. 244-67.
