# Supplementary material for: Improving Sleep Among Teachers: an Implementation-Intention Intervention
Source: Int J Behav Med. 2022 Mar 1;30(1):49–61. doi: 10.1007/s12529-022-10069-7 (PMC9879815; doi:10.1007/s12529-022-10069-7)
Supplement: Supplementary file 2 — Supplementary file2 (DOCX 31 KB) [file 12529_2022_10069_MOESM2_ESM.docx]

Electronic Supplementary Material 2:

*Reasons for sleeping less than intended*

___________________________________________________________________________________

Control group (N=35) Intervention group (N=34)

Reasons^a^ N^b^ % N^b^ %

___________________________________________________________________________________

Working too long 301 30.7 118 12.4

Social activities in the evening 103 10.5 54 5.7

Media consumption in the evening 91 9.3 28 2.9

Disturbing thoughts when going to bed 69 7.0 19 2.0

Too hot outside/inside 7 0.7 57 6.0

Problems falling asleep / staying asleep 41 4.2 28 2.9

Partner (sleep-wake-cycle, snoring) 27 2.8 14 1.5

Having a long way to work 25 2.6 11 1.2

Doing the chores in the evening 12 1.2 21 2.2

Appointments in the evening 11 1.1 6 0.6

Family (children, looking after parents) 10 1.0 30 3.2

Being sick 10 1.0 9 0.9

Physical activity/sports in the evening 8 0.8 9 0.9

Getting up early to go on short trips before work 0 0 10 1.1

___________________________________________________________________________________

*Intended sleep duration reached/woke up by myself 265 27.0 538 56.5*

___________________________________________________________________________________

Total 980 100 952 100

___________________________________________________________________________________

*Notes.*

^a^ Reasons reported in the daily diary if the intended sleep duration was not reached;

^b^ Number of nights reporting the reason during intervention and follow-up period.
